# Notes and News

**Published:** 1891-06-27

**Authors:** 


### Notes and News.

Collected and Communicated.

Sir Prescott Hewett, Bart., Serjeant-Surgeon to the Queen, died last Saturday at his residence near Horsham. Sir Prescott had been in failing health since the beginning of the winter, and while in a weak state, about a month ago, he was seized with an attack of influenza and congestion of the lungs, and this, with his other troubles, proved fatal.

The annual meeting of the Governors of the Dundee Royal Infirmary and Convalescent Home was held on the 8th inst., Mr. Thomas Maitland presiding. The Secretary read a very satisfactory account of the year's work, but the financial statement was not quite such pleasant reading, there having been an excess of expenditure over income of nearly £1,500. The Chairman moved that the governors should authorise the introduction of electric light in certain parts of the infirmary, and also of steam laundry machinery, at an estimated cost respectively of £285 and £700. This motion was unanimously adopted.

The annual report of the Salford Hospital Department shows that the working classes are recognising the benefits of hospital treatment to persons suffering from infectious diseases. Mr. John W. Mullen, the Medical Superintendent, states the sentimental objection to the fever hospital is rapidly disappearing, and there is a general feeling among the working people that even little children cannot receive that care and attention at home that they receive in the hospital, as a proof of which no less than forty-five infants, unaccompanied by their mothers, were admitted into the hospital last year. The total number of patients in 1890 was 991. In addition to the report, four charts are published showing the "incidence of disease as noted by the hospital department for the year, as compared with that for the eight years ending 1890."

A meeting of the General Committee in connection with the new Infirmary for Halifax was held on the 12th inst., for the purpose of receiving a report as to the plans. Mr. T. H. Morris said that the general principle of the accepted plans received the approval of the Executive Committee in October last, but there had remained many details to consider, chief among which was the method of heating to be adopted. The majority of the modern hospitals were found to be in favour of heating by steam, as being the simplest and least expensive. Engineers had been consulted with the result that they had received an offer to heat the Infirmary by steam for £2,500. The Committee had decided to abandon for the present the nurses' home, and also the isolation ward, and would erect for a commencement six wards, affording accommodation for twenty-two beds in each. The total amount of subscriptions promised up the present is £55,000, and the estimated cost of carrying out the plans is £50,000. Some members of the Committee objected to the adoption of the one-storey system, but these objections were over-ruled.

Eastbourne was gay with bunting and triumphal arches on Saturday last when the Prince and Princess of Wales and their daughters visited the town to open the new Convalescent Hospital for Children at Meads. The Royal party were received with great enthusiasm by the vast crowds which lined the route from the station to the hospital, which is a handsome red-brick building, facing the sea at the western end of the town. On arriving at the hospital an address was presented thanking the Princess for opening the new annexe. The Prince replied by a short speech, in which he said that it gave the Princess and himself great pleasure to further the good work; and after a prayer had been offered by the Bishop of Chichester, the Princess declared the hospital open.

This new hospital, of which the foundation-stone was laid in July last by the Duchess of Albany, is intended as a memorial to Harriet Brownlow Byron, founder of All Saints', Margaret Street, London. She had always greatly desired that the sufferings of little children should be specially alleviated, and on her death in 1887 the idea of making her unaccomplished work a practical testimony occurred to her friends. The new building, which has cost £10,000, will give accommodation for 130 beds. It is heated by steam and perfectly ventilated, while cheery day-rooms, covered play-ground, and comfortable dormitories will make it a pleasant home as well as a hospital.

With the object of attracting public attention and support to the Great Northern Central Hospital, a demonstration and church parade of amalgamated friendly, temperance, and trade societies took place last Sunday. The muster was in Blackstock Road, Finsbury Park, and when the time for moving off had arrived an imposing procession had formed. The procession wended its way, with banners flying, to St. Peter's Church, Dartmouth Park Hill, where Divine service was held. The Rev. J. F. Osborne preached a sermon specially appropriate to the occasion, and directed towards supplementing to a liberal extent the harvest reaped by the collecting boxes which had been vigorously worked *en route*.

On the invitation of the medical staff of the Central London Throat, Nose, and Ear Hospital, Mr. Emil Behnke, last Saturday, delivered a lecture on "Stammering." The lecturer dealt with a scientific method of grappling with stammering, in which the work of the teacher should be undertaken only after a medical opinion had been given that the patient was a fit subject for mental and elocutionary discipline, which could be of no avail in the presence of any physical cause for the impediment. Mr. Lennox Browne, the senior surgeon of the hospital, who presided, moved a cordial vote of thanks to the lecturer, which was carried by acclammation.

In common with all hostesses who desire their entertainments to be perfect successes, Bournemouth, the queen of the southern watering-places, is making ready for the guests she expects in the end of July. The British Medical Association now numbers 13,500 members, and though all invitations will not bring acceptances, still Bournemouth expects to have her hands pretty full with the guests who will hold their fifty-ninth annual meeting there. Dr. Roberts Thomson will preside over the whole as host, while Dr. Pye-Smith will undertake the medical section, Dr. Ward Cousins the surgical, Dr. Maury Deas the psychological, and Dr. Dickinson the pathological. With regard to the usual annual sermon, it will be preached by the Bishop of Winchester. The Corporation of the town have determined to make the event a memorable one, and have arranged for magnificent illuminations of the Pier, Boscombe Chine, and the Central Gardens on the night of July 28th Admirers of Bournemouth's beautiful cliffs and chines by day have little idea of their entrancing loveliness when illuminated; it is, therefore, no vain conclusion to draw that the fame of its healing breezes, odorous of pines, and the bracing air of the heights of Boscombe, its suburb will, as a result of the British Medical Association's visit, be spread even further and wider than they already are.

